# Linking market authorizations of medicines with disease burden in South Africa

**DOI:** 10.1186/s40545-021-00314-x

**Published:** 2021-04-01

**Authors:** K. Narsai, H. G. M. Leufkens, A. K. Mantel-Teeuwisse

**Affiliations:** Division of Pharmacoepidemiology & Clinical Pharmacology, Utrecht Institute for Pharmaceutical Sciences (UIPS), Utrecht University, Utrecht, the Netherlands

**Keywords:** Disease burden, Median registration timeline, HIV/AIDS, Tuberculosis, South Africa, SAHPRA

## Abstract

**Background:**

Sub-Saharan Africa is going through an epidemiological transition, including an impressive increase in non-communicable diseases. The introduction of medicines has not kept pace with the needs in developing countries. The objectives of this study were to (i) examine the correlation between the number of medicine approvals and disease burden and (ii) compare approval timelines of medicines with disease burden in South Africa in the period 2012–2017.

**Methods:**

The dataset was compiled from publicly available data on medicines registered in South Africa between 2012 and 2017. A correlation analysis was conducted to determine the level of alignment between the number and nature of medicines registered, as determined by the WHO ATC Classification and the Lancet Global Burden of Disease data. Median registration timelines were determined to assess whether medicines for diseases of higher burden were registered faster.

**Results:**

A total of 3059 registered medicines were included in the study, including 2779 generic medicines, 267 new chemical entities and 13 vaccines. There was a high level of alignment between the number of medicines registered to treat diseases with higher disease burden levels more effectively, except for lower respiratory tract infections and HIV/AIDS which showed less medicines registered as compared to expectations based on disease burden, respectively. HIV/AIDS showed a lower level of correlation with a much higher disease burden compared to number of medicines registered, but simultaneously also a much shorter median registration timeline (32 months) compared to the other disease areas.

**Conclusions:**

There was generally a high level of alignment between disease burden and number of medicines authorised, except for HIV/AIDS and lower respiratory tract infections. Regulatory authorities should continue to consider burden of disease data to ensure that public health needs are met.

## Background

The Lancet study on Global Burden of Disease showed that sub-Saharan Africa is going through a critical epidemiological transition in terms of disease burden in general. As countries increase their levels of development, communicable disease burdens seem to decline, life expectancies increase, but so will the burden of non-communicable diseases and injuries [1]. Overall, HIV/AIDS remains the leading cause of death in both South Africa and Kenya and out of 8 million deaths in 2015 in sub-Saharan Africa, 50% were due to malaria, HIV/AIDS, maternal and child health, anaemia and malnutrition. But, one-third were also due to cardiovascular disease and cancer [1, 2].

The issue of burden of disease has become top of mind even for many ordinary South Africans when this issue made local media headlines in 2019 referencing the Global Burden of Disease Study [3]. International attention was also drawn to the South African health system and its associated complex disease burden [4–7]. In a follow-up publication, Mayosi et al. referred to the shortcomings in measuring progress through consolidated and accurate public health information systems, even though various credible sources including the District Health Barometer, South African Health Review, StatsSA and others exist [8]. Even though significant progress has been made in the areas provision of treatment for HIV and tuberculosis in a short period of time through policy changes that led to the roll-out of large-scale antiretrovirals to HIV-infected patients, it is difficult to obtain population-based data that are accurate which shows progress in addressing burden of disease [9]. Similarly, a comprehensive programme for the management of tuberculosis (TB) has also been rolled out.

In the area of non-communicable diseases, there is increasing interest from many stakeholders [7]. Currently, the South African Medical Research Council is undertaking a second national burden of disease study and preliminary results show that there has been a decrease in the mortality rates of HIV/AIDS and TB, non-communicable diseases and injuries in South Africa for 2010 [9].

Having safe and efficacious medicines available for treatment is critical to the success of public health programmes and for managing disease burdens of countries. The global introduction of medicines has not kept pace with the needs in developing countries. One of the identified causes of delay in introducing new therapeutic options has been the constraints at the level of national regulatory approval for these medicines [10–13]. Medicine registration is a crucial phase in drug development as it permits the legal use of a medicine in a particular country [14]. National Medicines Regulatory Authorities (NMRAs) in Africa have for many years managed a broad range of activities with limited resources [15, 16]. Their focus has been on providing the local populations with a wide range of essential medicines, based on safety, efficacy and quality.

Globally, national regulatory approval is an important step often to guarantee widespread and sustainable long-term access to medicines. Regulatory approval is meant to serve a public good and remains essential for sustainable import, programmatic use and therefore optimising patient access to medicines [10]. As pharmaceutical innovation progresses, the challenge for regulators to advance regulatory approvals in a timely manner whilst ensuring patient safety will become increasingly challenging [17, 18]. South Africa is almost never in the first wave of countries in which companies seek registration for new medicines. However, South Africa is an active participant in international clinical trials on new treatments that are being developed for some of the top diseases that represent a high burden of disease in the country such as HIV/AIDS and cancer [19, 20]. Even so many of the treatments tested on patients during clinical trials conducted in South Africa do not eventually get registered in the country [21].

The premise of this study was to focus on diseases that showed the highest burden in South Africa, as per data obtained from the Lancet Global Burden of Disease statistics and then to look at the available medicines and vaccines to treat them. It is important to note that for these diseases many generic medicines are already available. This is very important to consider in the South African context where government policy favours the promotion and use of generic medicines [22, 23].

When South Africa transitioned to a democracy, as part of the overall health plan, amongst many initiatives to transform healthcare essential drug lists and standard treatment guidelines were developed and legislation was passed to create a new medicines regulatory authority [13, 24], the Medicines Control Council (MCC), which has since been transitioned to the South African Health Products Regulatory Authority (SAHPRA). This transition has been a huge undertaking and has taken immense energy and capacity of all stakeholders involved [13, 18, 25, 26]. Keyter et al. have highlighted the progress that has been made over the last couple of years in building a more public health centred and sustained regulatory system in South Africa [13].

The objectives of this study were therefore to (i) examine the correlation between the number of medicine approvals and disease burden and (ii) compare approval timelines of medicines with disease burden in South Africa in the period 2012–2017. The hypothesis was that diseases with higher levels of disease burden would correlate with higher numbers of registered medicines used to treat these diseases. A second hypothesis was medicines used to treat diseases with higher levels of disease burden would have shorter median registration timelines.

## Methods

### Data sources for medicines registered

Data on medicines registered in South Africa in the period 2012 to 2017 were included in the study. The dataset was compiled from publicly available data published by the Medicines Control Council (MCC) listing all medicines registered on their website (www.mccza.com). The MCC was the national regulatory agency in South Africa during the study period [13].

The dataset was validated by verifying the lists of registered medicines with those published in the Government Gazettes covering the same time periods. The dataset was re-validated with the most updated set of data in May 2020, as published by the new regulatory agency, South African Health Products Regulatory Agency (SAHPRA) on their website (www.sahpra.org.za) [27].

The timeframe was chosen for methodological reasons to ensure a reasonable timeframe of 5 years and based on availability of data from the regulatory authority and Government Gazette at the time of data collection. The cutoff for the timeframe was 2017 which aligned with the transitioning of the old regulatory authority, the MCC to the new South African Health Products Regulatory Authority (SAHPRA)*.*

The following categories of products were excluded from the dataset: veterinary products, nontherapeutic agents such as solvents or contrast media, complementary medicines, lozenges, gum, vitamins and medical devices, including combination medical devices, such as hormone releasing IUDs.

### Data extraction and classification

The unit of analysis was the market authorisation or medicine registration information for each medicine registered, which included the following data fields: application number, company name, proprietary name of medicine, international nonproprietary name (INN). Each formulation of the same medicine was regarded as a separate medicine.

The WHO Anatomical Therapeutic Chemical (ATC) classification system and the Defined Daily Dose (DDD) classification for each medicine was established through an online search of the active pharmaceutical ingredient (API) of each medicine on the WHO ATC/DDD Index website (https://www.whocc.no/atc_ddd_index/). The second level of WHO ATC/DDD classification was used for the analysis which describes either the pharmacological or therapeutic groups [28].

### Determining the disease burden

The Lancet Global Burden of Disease Study (GBD) is the most up-to-date and comprehensive burden of disease data available globally and provides the most comprehensive data on morbidity, mortality, underlying risk factors, including behavioural factors across geographies, age groups and gender [1, 4].

We chose to use data from 2017 and the top 10 causes of mortality for South Africa from the GBD database for all ages and both sexes. Categories related to trauma which would require non-medicine interventions were eliminated, leaving 8 conditions for the analysis which were limited to conditions classified as communicable and non-communicable diseases. For more information on this data source, see http://ghdx.healthdata.org. Mortality data were chosen for the study over disability adjusted life years (DALYs) as mortality data were considered more applicable for the South African context [9]. The percentage disease burden values were compared for 2012 and 2017 and no significant differences were found. Based on this finding, percentage disease burden data for 2017 were used for the purposes of analysis for this study.

### Data analysis

The number of NCEs registered per WHO ATC/DDD classification was determined over the study period to determine any upward or downward trends in total numbers registered. The correlation analysis was done on two levels of the registered medicines data: (1) on the total number of medicines registered, including duplicate medicines if generics contained the same (API) and (2) on the total number of medicines registered with unique (API) which correlated with the disease burden data.

High levels of correlation were defined as ≤ 5% difference between the values for percentage disease burden (a) and percentage medicines registered with a unique API (b) or difference between the values for percentage disease burden (a) and percentage total medicines registered (c). Low levels of correlation were defined as >  5% of these values. The percentage disease burden values were taken directly from the GBD database and the percentage medicines registered were taken from the dataset analysed for this study (*n* = 3059). The percentage medicines with a unique API was defined as percentage of medicines containing a unique API vs total medicines registered which are used to treat the top 8 diseases, as defined in the GBD database.

Median registration timelines were calculated using the application number for medicines used to treat the top 8 diseases to determine whether those used to treat diseases with higher levels of disease burden would be registered faster. Since the MCC did not make actual registration timeline data available, median registration timelines were calculated using the midpoint of the year of submission normalised to 30 June of each submission year based on the assumption that submissions could be made at any time of a submission year.

## Results

A total of 3059 registered medicines were included in the study: 2779 generic medicines, 267 new chemical entities (NCEs) and 13 vaccines, which were registered by the MCC during the period 2012 to 2017. Overall, generic medicines showed longer median registration timelines than NCEs, and timelines increased for both over time. The registration timelines for NCEs and generic medicines were similar across the years 2012, 2013, 2015 and 2016 (Table 1). In 2014 and 2017, however, NCEs median registration timelines were significantly shorter than those for generic medicines (2014: 49 months vs 62 months, 2017: 47 months vs 74 months). Generic medicines were registered predominantly by international generic manufacturers (*n* = 1516), while local generic manufacturers accounted for 1074 medicines registered and multinational R&D manufacturers accounted for 469 medicines registered.

**Table 1:** General characteristics—registered medicines

Year	2012	2013	2014	2015	2016	2017	Total
NCEs registered	76	54	43	18	19	57	267
Generics registered	600	549	477	403	423	327	2779
Vaccines registered	3	1	2	3	1	3	13
Total number of medicines registered	679	604	522	424	443	387	3059
Median time to approval in months (total)	48	47	62	64	69	72	60*
Generics median time to approval (months)	48	47	62	64	69	74	61*
NCEs median time to approval (months)	48	51	49	66	69	47	55*
Vaccines median time to approval (months)	35	-	41	64	-	35	

WHO/DDD classification Total number of medicines registered (NCEs registered)^a^	2012	2013	2014	2015	2016	2017	NCEs registered
A (alimentary tract and metabolism) (*n* = 195)	53 (14)	34 (3)	58 (4)	18 (6)	14 (3)	18 (8)	38
B (blood and blood forming organs) (*n* = 51)	16 (4)	13 (10)	7 (1)	3 (0)	7 (0)	5 (0)	15
C (cardiovascular system) (*n* = 577)	105 (18)	144 (2)	87 (0)	82 (0)	92 (2)	67 (10)	32
D (dermatologicals) (*n* = 32)	6 (1)	7 (0)	6 (0)	11 (0)	2 (0)	–	1
G (genitourinary system and sex hormones) (*n* = 104)	22 (3)	10 (6)	19 (1)	21 (4)	14 (3)	18 (1)	18
H (systemic hormonal preparations, excl. sex hormones and insulins) (*n* = 42)	6 (1)	2 (1)	6 (3)	8 (0)	14 (1)	6 (6)	12
J (anti-infectives for systemic use) (*n* = 634)	149 (3)	114 (9)	110 (5)	91 (6)	110 (1)	60 (0)	24
L (antineoplastic and immunomodulating agents) (*n* = 353)	89 (21)	49 (10)	50 (17)	40 (1)	65 (7)	60 (11)	67
M (musculoskeletal system) (*n* = 117)	8 (1)	22 (2)	20 (1)	24 (0)	18 (0)	25 (0)	4
N (nervous system) (*n* = 727)	154 (6)	177 (10)	127 (8)	97 (0)	84 (1)	88 (12)	37
P (antiparasitic products, insecticides and repellents) (*n* = 5)	–	2 (0)	2 (0)	–	–	1 (1)	1
R (respiratory system) (*n* = 183)	65 (2)	29 (0)	23 (0)	19 (0)	16 (0)	31 (8)	10
S (sensory organs) (n = 30)	5 (2)	1 (0)	3 (0)	6 (0)	7 (1)	8 (0)	3
V (various) (*n* = 9)	1 (0)	–	4 (3)	4 (3)		–	6
**Medicines registered by company type**							
Local generic manufacturer	216	224	145	176	175	138	1074
International generic manufacturer	334	308	283	185	222	184	1516
Multinational R&D pharma company	129	72	94	63	46	65	469

^a^Average of medians

**Fig. 1:**
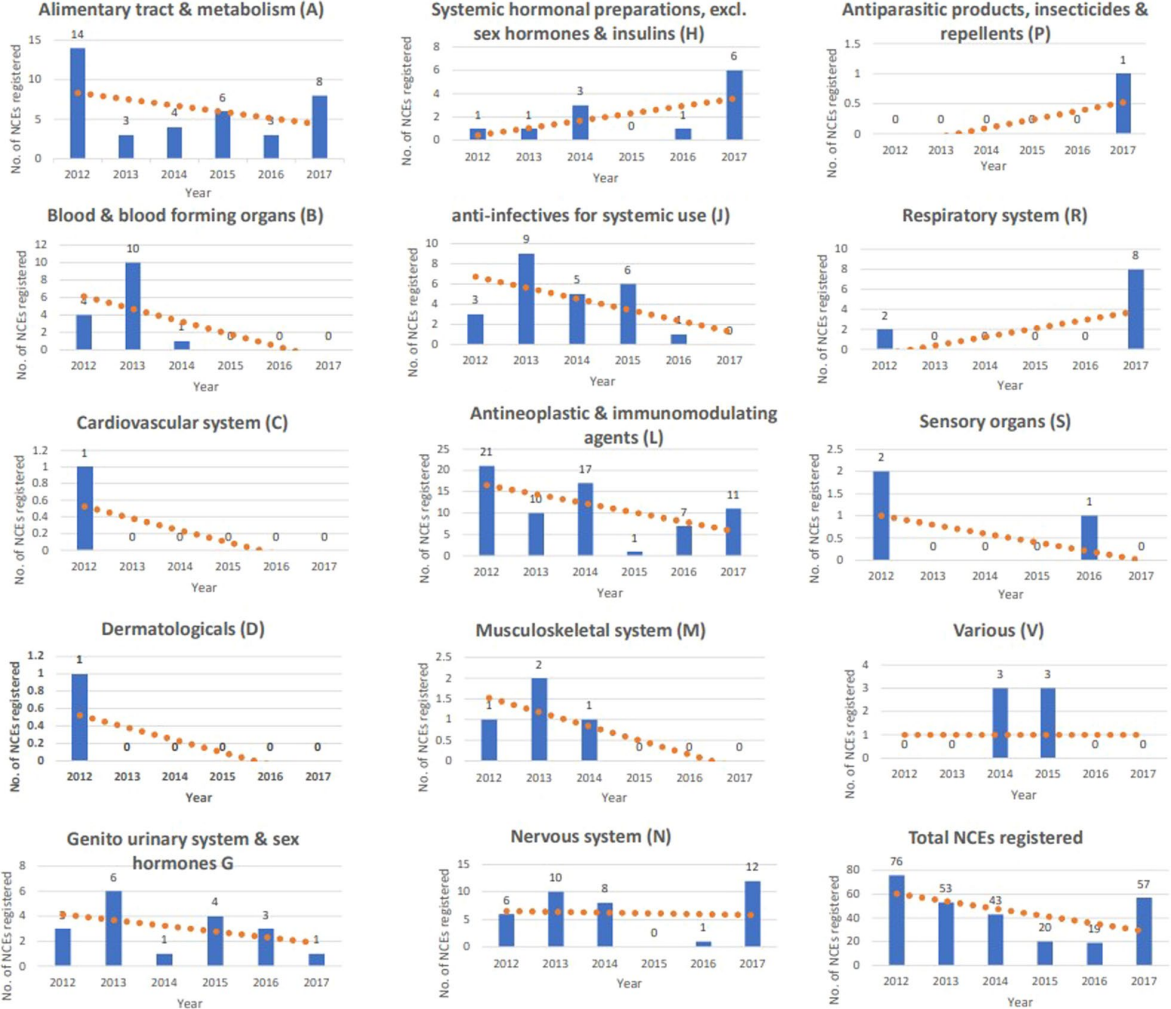
Number of NCEs registered over study period (2012–2017) by WHO classification [28]

The overall number of NCEs declined over the study period and then showed an increase in 2017 (Fig. 1). Most WHO/ATC classifications showed an overall downward trend in the number of NCEs registered over the study period, while the category “Lower Respiratory Diseases” showed an upward trend. The trend for medicines used for “Other dementias” was flat, while the results for other categories were difficult to interpret, since the number of data points were limited (Fig. 1).

**Fig. 2:**
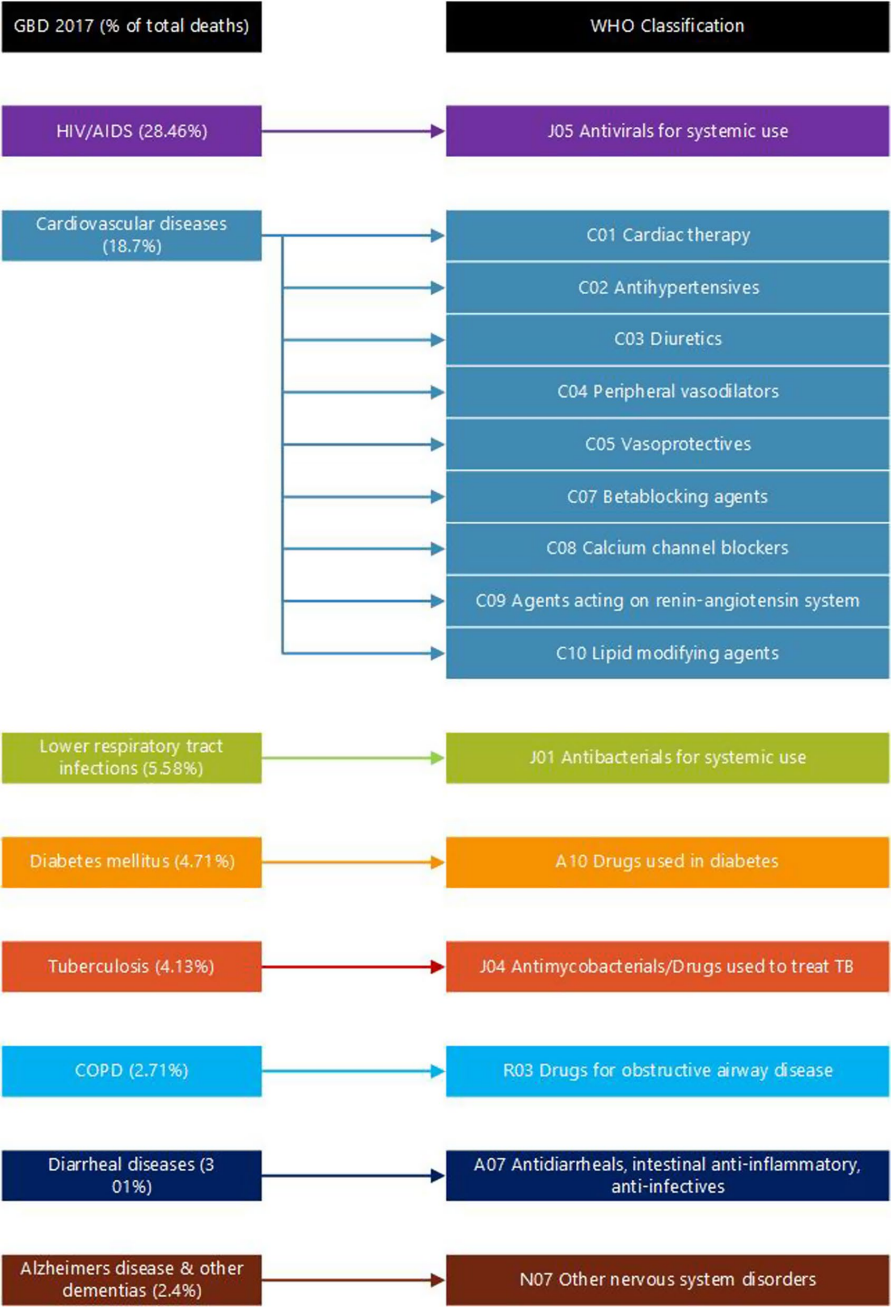
Burden of disease and related medicines [1]

South Africa still suffers from the challenge of a high burden of disease due to infectious diseases, in particular, HIV and TB. HIV/AIDS remains the highest cause of mortality with 28.46% of total of deaths (Fig. 2). Non-communicable diseases are also fast on the rise with cardiovascular diseases making the list of the top 10 conditions in the cause of death statistics data according to the GBD data analysis. Cardiovascular diseases accounted for 18.7% of the burden of disease showing the high impact of non-communicable diseases on the total burden of disease. Similarly, diabetes mellitus (4.71%), chronic obstructive pulmonary disease, COPD (2.71%) and Alzheimer’s disease and other dementias (2.4%) also featured in the top 10 leading causes of death in South Africa.

A total of 1476 medicines (91 NCEs and 1385 generics) were registered during the study period which correlated with the top 8 diseases. When looking at medicines with unique active pharmaceutical ingredients (APIs) whether generic or NCEs, 192 unique medicines remained. There was a high level of alignment between the number of medicines registered to treat diseases with higher levels of disease burden in order to address the disease burden.

For HIV/AIDS the high burden of disease did not correlate with a high number of medicines registered during the study period (correlation = 24), but did correlate with a shorter median registration timeline (32 months) (Table 2). Throughout the study period, only 17 medicines were registered for the treatment of TB, one being bedaquiline for the treatment of MDR-TB, which was registered in 2014. Eleven linezolid generics were registered between 2016 and 2017, which is a listed treatment on the treatment guidelines for TB in South Africa [24]. Median registration timelines for TB medicines were also shorter than for other diseases (Table 2).

**Table 2:** Proportionality analysis of number of medicines registered vs top 8 disease burdens

TOP 8 diseases	% Disease burden (a)	Medicines registered with unique APIs				Total medicines registered			
		n	% (b)	Correlation (a, b)	Median registration time (months)	n	% (c)	Correlation (a–c)	Median registration time (months)
Cardiovascular	29	49	25	4	57	580	38	**− 9**	56
COPD	4	13	7	− 3	70	99	7	− 3	39
Diabetes mellitus	7	12	6	1	50	49	4	3	48
Diarrheal diseases	4	3	2	2	57	9	1	3	62
HIV/AIDS	40	51	26	**14**	32	233	16	**24**	32
Lower respiratory infections	8	39	20	**− 12**	60	327	22	**− 14**	63
Other dementias	3	18	9	**− 6**	67	162	11	**− 7**	56
Tuberculosis	6	10	5	1	39.5	17	1	5	40

High levels of correlation were defined as ≤ 5% difference between the values for % disease burden (a) and % medicines registered unique API (b) or difference between the values for % disease burden (a) % total medicines registered (c). Low levels of correlation were defined as > 5% of these values

The number of medicines registered which are used to treat non-communicable diseases was also high as expected based on burden of disease in the following categories: N (nervous system; *n* = 727), C (cardiovascular disease; *n* = 577), L (antineoplastic and immunomodulating agents; *n* = 353) and A (alimentary tract and metabolism; *n* = 195). A high number of oncology medicines, L (antineoplastic and immunomodulating agents; *n* = 353) were registered throughout the study period with the highest in 2013 (*n* = 177). Median registration timelines were also shorter for COPD. Even though cardiovascular disease showed a high level of disease burden, the median registration timeline was longer than for other diseases with lower levels of disease burden (Table 2).

## Discussion

The hypothesis was that diseases with higher levels of disease burden would correlate with higher numbers of registered medicines used to treat these diseases and that medicines used to treat diseases with higher levels of disease burden would have shorter median registration timelines. This would enable treatment of these diseases more effectively, to serve the public health needs of the South African population.

Based on the data analysis, high levels of correlation were shown for the following disease areas: cardiovascular disease, COPD, diabetes mellitus, diarrheal diseases and tuberculosis. These diseases showed a high burden of disease as well as a high number of registered medicines during the study timeframe. HIV/AIDS and lower respiratory infections, however, showed a lower correlation between the number of medicines registered and the level of disease burden.

There was a much higher number of generic medicines registered over the study period (*n* = 2779), which demonstrates positive alignment with the government’s overall strategy to promote the use of generic medicines [11, 12, 23, 29]. However, the median time to registration observed increased over the study period (2012: 48 months vs 2017: 74 months) and therefore it can be concluded that these medicines are not readily available on the market in a timely manner. Local generics manufacturers did not benefit from any prioritisation in terms of shorter review times or expedited registrations. In addition, generics have longer median registration timelines vs NCEs.

Even though there were a high absolute number of ARVs registered, the disease burden for HIV/AIDS was proportionally much higher. This could be explained by the extensive ARV roll-out programme that has been is already in place, which includes provision of ARVs to HIV positive patients and is the largest of its kind in the world and also the fact that the HIV/AIDS disease burden of the country remains high [30].

Another major public health threat lies in the area of TB where only a few medicines were registered (*n* = 17). A significant lag time was observed between the global registration and the South African registration of bedaquiline [10]. In this disease area, there was a low level of alignment with public health need. This is aligned to recent research that showed that global introduction of TB products has not kept pace with the need due to delays in national regulatory approvals [10]. This phenomenon is not unique to South Africa.

In the dataset, there were a significant number of oncology medicines registered (*n* = 353), which is reflective of the shift of disease burden towards non-communicable diseases. Even though cancer did not feature in the top 10 causes of death according to the GBD study, another study points to the increasing burden of disease related to cancer in South Africa [31]. Lung cancer, cervical cancer and oesophageal cancer are the three deadliest cancers in South Africa, accounting for 19,160 deaths in 2015, according to a new analysis of 32 cancer groups in 195 countries or territories. In 2015, there were 114,091 new cancer cases in South Africa and 58,237 deaths in total [32].

Recent studies showed that the majority of novel medicines are indicated for non-communicable diseases (NCDs) such as cancers, cardiovascular and neuropsychiatric disorders. NCD medicines are also the largest contributor to generics globally and account for 66% of the low income countries medicines market [11, 14, 33]. Recent literature shows the rise of diabetes in South Africa as a leading cause of morbidity and mortality and the data showed that a proportionate number of medicines were registered for the treatment of diabetes mellitus (*n* = 49), with fewer NCEs (*n* = 19) registered [34].

Of concern is the total number of NCEs registered over time, which showed a decline in most ATC classifications. The reasons for this decline could be numerous, including companies not filing for registration, withdrawal of applications or lengthy registration timelines but this could not be determined in the scope of this study. However, an increase in the number of registrations of NCEs was observed in 2017 with a slightly reduced median registration time. This could be related to the new regulatory authority becoming fully operational over this time period [26]. It remains to be seen if this trend will continue into the future.

In the past, the MCC outsourced regulatory review processes to external consultants to assess data provided in the applications for registration of medicines, vaccines, biologicals, veterinary and complementary medicines who were not bound by any performance agreements. Since the transition to SAHPRA, a concerted effort has been made to build in-house capacity [29, 35].

The median registration timelines for NCEs was notably shorter than those for generic medicines. Although median registration times are important for new medicines that represent significant therapeutic improvements, the majority of medicines registered in South Africa are generics. This is in line with the broader government objectives of promoting the use of generics, in particular, locally manufactured generics for both cost saving and industrial policy objectives [11, 23].

However, these policy objectives cannot be realised if median registration timelines are excessively long. In fact, local manufacturers have cited this as a challenge to doing business in this area [36, 37]. On the other hand, having too many generics entering the regulatory system can also have the potential of clogging the system and increase the median registration timelines and dilute the benefits of availability and cost saving.

A recent study which investigated the South African tendering system for medicines to assess the impact on prices and market concentration, included analysis on antibiotics and found that even though this market remained highly or moderately competitive over a 14-year period (2003–2016), there was a decline in the number of companies bidding for tenders possibly due to the lengthy registration timelines experienced in getting medicines to market, with applications for new medicines taking up to 3 years (36 months). This timeline is shorter than the findings of this study which showed an average median registration timeline of 55 months for NCEs and 61 months for generics. The authors cite regulatory barriers to market entry as a threat to competition and lack of coordination between the issuing of tenders and the registration of medicines which can lead to medicines being unduly excluded from tenders, therefore not only weakening competition, but also reducing the availability of important medicines in the public health system [38].

Our study provides, for the first time, an analysis of whether the medicines registered in South Africa are aligned with the burden of disease in South Africa. The added value of this study is to provide evidence that can guide policymaking in South Africa to ensure that appropriate medicines are available to treat the disease burden in South Africa and to ensure policy alignment between different regulatory authorities/government departments that may have interdependencies to meet their respective priorities.

Since the establishment of the new regulatory authority, SAHPRA, allows for even more independence in terms of financing, organisational structures, operational processes and scope of functions, collaboration with government, private sector and civil society actors will become increasingly critical to ensure alignment with the shifting disease burden of the country. As an example, the disease management programmes implemented by the Department of Health would be dependent on having appropriate medicines available, which are manufactured by the private sector and acceptable to civil society. A Bill is being finalised in South to establish a National Institute of Public Health, which will amongst other functions also be responsible for disease burden data [39]. This entity will go a long way in creating transparency and accuracy in public health data upon which sound decisions can be made by policymakers.

### Limitations of the study

The study was limited to the data available in the public domain. Therefore, there is a possibility of missing data which the regulatory authority have not made available in the public domain. Data validation was conducted between the data published by the MCC and that published in the Government Gazette. No medicines were identified in the Gazettes that were not on the agency’s list. However, it was found that not all the data published by the MCC over the time period were published in the Government Gazette. In terms of the ATC/DDD classification, there is currently no specific classification for medicines used for the treatment of Alzheimer’s disease.

Only median registration timelines could be calculated based on the midpoint of each submission year based on the fact that the exact submission dates were not available from the MCC. It is acknowledged that there are many other factors that may influence the frequency of approvals by disease areas; e.g. if companies do not innovate/manufacture and/or submit applications for marketing authorisations, medicines will not get approved irrespective of available regulatory resources. Most research and development activities take place outside of South Africa, although South Africa is very actively involved in clinical research [19, 20]. The study only evaluated approvals and therefore does not take into consideration applications for registration of medicines which may have been removed by the applicant company or may have been rejected by the MCC. It should be noted that the number of medicines registered does not necessarily correlate with their therapeutic value, i.e. many medicines of marginal therapeutic value could be registered for a particular disease. In addition, in the context of South Africa’s policy to promote the use of generic medicines, multiple medicines with the API are registered by the authority. Finally, even though there were no significant changes or differences in disease burden data between 2012 and 2017, the use of the 2017 burden of disease data for the purposes of analysis could be considered as a limitation as fluctuations during the study period were not considered in the analysis.

## Conclusion

In conclusion, this study showed higher levels of alignment between medicines registered and disease burden for most of the high prevalence diseases except for lower respiratory tract infections and HIV/AIDS which showed less medicines registered as compared to expectations based on disease burden, respectively. Regulatory authorities should continue to consider burden of disease data to ensure that public health needs are met.

## Data Availability

The data that support the findings of this study are available from the corresponding authors upon reasonable request.

## Abbreviations

WHO/DDD: World Health Organization/Daily Defined Dose
SAHPRA: South African Health Products Regulatory Authority
MCC: Medicines Control Council
GBD: Global Burden of Disease
